# High prevalence of rare *FBLIM1* gene variants in an Italian cohort of patients with Chronic Non-bacterial Osteomyelitis (CNO)

**DOI:** 10.1186/s12969-020-00447-4

**Published:** 2020-07-10

**Authors:** Adamo Pio d’Adamo, Anna Monica Bianco, Giovanna Ferrara, Martina La Bianca, Antonella Insalaco, Alberto Tommasini, Manuela Pardeo, Marco Cattalini, Francesco La Torre, Martina Finetti, Clotilde Alizzi, Gabriele Simonini, Virginia Messia, Serena Pastore, Rolando Cimaz, Marco Gattorno, Andrea Taddio, for the Italian Pediatric Rheumatology Study Group

**Affiliations:** 1 University of Trieste, Trieste, Italy; 2 Institute for Maternal and Child Health - IRCCS “Burlo Garofolo”, Via dell’Istria 65/1, 34100 Trieste, Italy; 3 Department of Pediatric Medicine, Division of Rheumatology, Bambino Gesù Children’s Hospital, Rome, Italy; 4 Pediatric Clinic University of Brescia and Spedali Civili of Brescia, Brescia, Italy; 5 Pediatric Rheumatology Center, Pediatric Unit, “Giovanni XXIII”, Pediatric Hospital, Bari, Puglia Italy; 6 Centro Malattie Autoinfiammatorie e Immunodeficenze, IRCCS “G. Gaslini”, Genoa, Italy; 7 ”G. Di Cristina”Children’s Hospital, Palermo, Italy; 8 Pediatric Rheumatology Unit, AOU Meyer, University of Florence, Florence, Italy; 9 Azienda Socio Sanitaria Territoriale (ASST) G.Pini and University of Milan, Milan, Italy

**Keywords:** Chronic non-bacterial osteomyelitis, *FBLIM1* gene, Bone sterile inflammation, Autoinflammatory disease

## Abstract

**Background:**

*FBLIM1* gene has been recently demonstrated to be involved in the pathogenesis of bone sterile inflammation. The aim of the study is to evaluate the prevalence of *FBLIM1* gene variants in a cohort of 80 Italian patients with Chronic Non-bacterial Osteomyelitis (CNO).

**Methods:**

The coding regions of *FBLIM1* gene were sequenced in a cohort of 80 patients with CNO using DNA extracted from blood lymphocytes, and PCR products were sequenced. Only rare (global MAF < 2%), coding variants detected were considered. Clinical evaluation of patients with rare variants and those without was performed. Fisher’s exact test was used to compare categorical and ordinal data, and Student’s t-test was used to analyze continuous data.

**Results:**

Eighteen out of 80 patients (~ 22%) presented at least one rare coding variant in *FBLIM1*. Eight patients presented a variant never associated before with CNO. All patients presented classical features of CNO and no statistical difference between patients with presence of *FBLMI1* variants and those without were found in terms of clinical manifestation, treatment, and outcome.

**Conclusion:**

Considering the high frequency of rare variants in our CNO cohort, our data seem to confirm a possible role of *FBLIM1* in the pathogenesis of CNO suggesting that CNO is a disorder of chronic inflammation and imbalanced bone remodeling.

## Background

Chronic Non-bacterial Osteomyelitis (CNO) is a rare inflammatory disorder that is characterized by onset of pain, local bone expansion and radiological findings suggestive of osteomyelitis, usually at multiple sites, not related to an infectious disease [1]. Although its pathogenesis remains still unknown, there is consensus about the hypothesis that CNO might be a genetic disease within the spectrum of autoinflammatory disorders [2–4]. Although the existence of genes contributing to sporadic CNO has been proposed, their identification is still missing [5].

Cox et al have recently demonstrated that *FBLIM1*, a gene that codes for a protein involved in the regulation of bone remodeling, could be involved in the pathogenesis of sterile bone inflammation. The authors, via whole-exome sequencing, detected a homozygous mutation in the filamin-binding domain of *FBLIM1* in an affected child with consanguineous parents. They also sequenced *FBLIM1* in 96 subjects with CNO and found a second patient with a distinct frameshift variant [6].

On this basis, we sequenced the *FBLIM1* gene in a cohort of Italian patients with CNO and correlated the results with clinical manifestations.

## Methods

This is a multicenter observational study. In the absence of validated international diagnostic criteria, we used the proposed Jansson criteria for CNO diagnosis [7]. The clinical and radiological data were entered into a customized and anonymized database and considered as variables for correlation analysis. Blood samples were collected from patients affected by CNO from 7 Italian rheumatology centers: IRCCS Burlo Garofolo, Trieste; Spedali Civili, Brescia; Anna Meyer Children’s Hospital, Florence; Bambino Gesù Children’s Hospital, Rome; G. Di Cristina Children’s Hospital, Palermo; “Giovanni XXIII” Pediatric Hospital, Bari and IRCCS Giannina Gaslini, Genoa. Written informed parental consent, according to the approved protocol of the IRCCS “Burlo Garofolo” ethics committee (n°27/14) was obtained for genetic analysis. Blood collected for DNA analysis was taken specifically for this study during a routine blood sampling. After standard genomic isolation, DNA was used for Sanger sequencing on entire coding and flanking regions of *FBLIM1*. All sequences were analyzed with codon code aligner software (V.7.1.1 version). Only rare (global MAF < 2%), coding variants detected were considered. To predict if each variation could be harmful for the protein function, we used the following softwares: Polyphen2 (http://genetics.bwh.harvard.edu/pph2/); SIFT (http://sift.jcvi.org); MUTATION ASSESSOR (http://mutationassessor.org/r3/); Human Splicing Finder (http://www.umd.be/HSF/) and TRANSFAC (http://gene-regulation.com/pub/databases.html#transfac). Controls were taken from the gnomAD, a worldwide database of hundreds of thousands of individuals with no manifested pathologies, stratified by regions of origin.

Clinical evaluation between patients with and without rare variants was performed. Fisher’ s exact test was used to compare categorical and ordinal data, and Student’s t test was used to analyze continuous data.

## Results

Eighty patients diagnosed with CNO were enrolled, 52 females (65%) and 28 males (35%). Bone pain was the most common clinical complaint; median age at onset was 9 years (range 4–14 years). We found a median number of six bone localizations per patient; 9 patients had a single lesion. Most lesions were located to the long bones (65%), pelvis (48%) and spine (37%). Three patients presented skull lesions (they underwent bone biopsy to exclude malignancy). Five patients (5.8%) had a first-degree relative affected by an autoimmune disorder. Thirty patients (35%) had a comorbidity; among these, 18 (22.5%) had skin manifestations such as psoriasis, pustulosis or severe acne, 4 (5%) had inflammatory bowel disease. No consanguinity nor family history for CNO was reported.

Sanger sequencing was conducted in all patients. 18 out of 80 (22,5%) patients presented at least one rare (≤ 0.02) variant in *FBLIM1* gene. In particular, 15 patients presented 1 rare variant; 2 patients presented 2 rare variants and 1 patient had 5 rare variants. In total, 11 rare variants were found (7 out of 11 variants presented a MAF ≤ 0.01).

Table 1 displays the variants /SNPs identified. 5 out of 11 variants were in the coding regions and 6 were intronic. Among the SNP in the coding region 3 are missense and 2 synonymous variants. Two of the missense (Arg38Gln and Gly311Arg) and one of the intronic variants (c.250 + 32 C > A) have been previously reported in CNO by Cox et al [9]. The other eight variants have never been associated with CNO.

**Table 1:** Rare variants of *FBLIM1* gene identified in 18 patients affected by Chronic Non-Bacterial Osteomyelitis

	rs	Change	Amino acid changes	MAF	Variant	Patients
**1**	rs540511146	c.65G > A	p.Arg22His	A = 0.00011	missense	P12
**2**	rs201006671	c.-20-49G > T	N/A	T = 0.0033	intron	P18
**3**	rs76050903	c.-20-48C > A	N/A	A = 0.0033	intron	P18
**4**	rs146575757^a^	c.113G > A	p.Arg38Gln	A = 0.00391	missense	P18
**5**	rs61733331	c.222G > A	p.Pro74=	A = 0.00675	synonymous	P18
**6**	rs766409425	c.541 + 13G > A	N/A	A = 0.00003	intron	P7
**7**	rs187479896	c.717 + 14A > G	N/A	G = 0.00083	intron	P13
**8**	rs41310367 ^a^	c.250 + 32C > A	N/A	T = 0.0191	intron	P3; P11
**9**	rs140170023	c.447G > A	p.Ala149=	A = 0.01200	synonymous	P2, P3, P4, P5, P6, P8, P9, P10, P13, P15
**10**	rs114077715 ^a^	c.931G > A	p.Gly311Arg	A = 0.01955	missense	P18; P16
**11**	rs144567113	c.718-29C > T	N/A	T = 0.01515	intron	P1; P14; P17

^a^variants already described [8]

Patient P18 harbored 5 *FBLIM1* variants: two missense variants (rs146575757 Arg38Gln and rs114077715 Gly311Arg), two intronic (rs201006671 and rs76050903) and one synonymous (rs61733331) variant. By parental sequencing we verified that the child inherited from his mother the allele with the missense Arg38Gln, the two intronic and the synonymous variant while he inherited from his father the allele with the only Gly311Arg missense variant, found also in heterozygosis in P16. In patient P13 we identified in homozygosis the intronic variant rs187479896 and in heterozygosis the rs140170023, a synonymous variant present also in heterozygosis in other 9 patients (P2, P3, P4, P5, P6, P8, P9, P10, P13 and P15). In patient P3, in addition to the synonymous variant, the SNPs rs41310367 was also detected in heterozygosis. An intronic heterozygous variant was identified also in P11. Finally, P12 carried the missense variant Arg22His (rs540511146), P7 the variant rs766409425 and patients P1, P14, and P17 the intronic variant rs144567113.

All the 11 identified variants, especially those never described before and so orphaned of functional studies were analyzed “in silico” using specific software; results are shown in Table 2. Moreover, according to dbSNP, all the variants identified are extremely rare in healthy controls examined (supplementary table), thus enforcing their possible pathogenic roles.

**Table 2:** The in-silico analysis of the 11 FBLIM1 gene variants

NonSynonymous		Global MAF	In Silico Analysis		
**rs**	**CDS/AA**	**Allele/MAF**	**PP2**	**SIFT CADD**	**Mutation Assessor**
rs540511146	c.65G > A; p. R22H	A/0.00011	PD	D LB	Medium
rs146575757	c.113G > A; p. R38Q	A/0.00391	B	T B	Low
rs114077715	c.931G > A; p. G311R	A/0.01955	B	D LB	No, data
Synonymous		Global MAF	In Silico Analysis (HSF)		
**rs**	**CDS/AA**	**Allele/MAF**	**ESS**	**ESE**	**splicing site**
rs61733331	c.222G > A; p. Pro74Pro	A/0.00675	New ESS Site	ESE site broken	NASS
rs140170023	c.447G > A; p.Ala149Ala	A/0.01200	New ESS Site		NASS
Intronic Variants		Global MAF	In silico analysis		
**rs**	**SNVs**	**Allele/MAF**	**New Site**	**Broken Site**	**TRANSFAC**
rs201006671	c.-20-49G > T	T/0.0033			Broken site for ENKTF-1
rs76050903	c.-20-48C > A	A/0.0033			Broken site for ENKTF-1
rs766409425	c.541 + 13G > A	A/0.00003	SF2/ASF	SRp40	
rs187479896	c.717 + 14A > G	G/0.00083			
rs41310367	c.250 + 32C > A	T/0.0191		SF2/ASF/SRp55	
rs144567113	c.718-29C > T	T/0.01515			

Reference SNPs: rs; coding sequence/aminoacids: CDS/AA; Polyphen-2: PP2

Sorting Intolerant from Tolerant: SIFT; Combined Annotation Dependent Depletion: CADD

Probably Damaging: PD; Deleterious: D; Benign: B; Tolerated: T

Minor Allele Frequency: MAF; Exonic Splicing Silencer: ESS; Exonic Splicing Enhancer: ESE

All patients presented classical features of CNO and no statistical differences between patients with or without *FBLIM1* variants were found in terms of gender prevalence, positive family history, age at onset, number of sites involved, presence of fever, arthritis and skin involvement as well as remission at the end of follow-up (Table 3).

**Table 3:** Clinical presentation, laboratory data, treatment and outcome of CNO patients cohort divided following presence or absence of FBLIM1 variant. § 9 patients were lost at last follow-up

	Patients without FBLIM1 variants (62 patients)	Patients with FBLIM1 variants (18 patients)	P
**Gender female**	41 (66%)	11 (61%)	0,78
**Median Age at onset**	9.5 y (range 1.5–16)	8.9 y (range 1,5–12)	0,97
**Clinical data**			
Fever	30 (48%)	4 (22%)	0,06
Swelling	11 (18%)	7 (38%)	0,1
Pain	60 (96%)	15 (83%)	0,07
**Sites**		8.9 (median)	
Long bones	46 (74%)	13 (72%)	1,0
Pelvis	23 (37%)	11 (61%)	0,1
Column	22 (35%)	5 (27%)	0,78
Clavicle	16 (26%)	5 (27%)	1,0
Chest and ribs	24 (39%)	3 (16%)	0,09
Foot and hands	15 (24%)	4 (22%)	1,0
Mandible	11 (18%)	1 (5%)	0,28
Skulls	3 (5%)	0 (0%)	1,0
**Skin involvements**	11 (18%)	4 (22%)	0,73
**Bowel involvements**	3 (5%)	1 (6%)	1,0
**Renal involvements**	2 (3%)	0 (0%)	1,0
**Laboratory data**			
Elevated CRP	32 (52%)	14 (77%)	0,06
Elevated ESR	41 (66%)	13 (72%)	0,78
**Treatment**			
NSAIDs	55 (89%)	17 (94%)	0,67
Steroids	20 (32%)	6 (33%)	1,0
MTX	12 (19%)	3 (16%)	1,0
Sulfasalazine	10 (16%)	3 (16%)	1,0
Adalimumab	3 (5%)	1 (5%)	1,0
Etanercept	8 (13%)	2 (11%)	1,0
Infliximab	3 (5%)	2 (11%)	0,31
Anakinra	9 (15%)	3 (17%)	1,0
Neridronate	2 (3%)	1 (6%)	1,0
Pamidronate	29 (47%)	8 (44%)	1,0
**Outcome at last follow up§**			
Remission without therapy	32 (52%)	5 (28%)	0,11
Remission on therapy	18 (29%)	5 (28%)	1,0
Active disease	6 (10%)	5 (28%)	0,11

## Discussion

CNO is a rare pediatric autoinflammatory bone disease and several factors such as family pedigree and syndromic monogenic forms of CNO suggest a strong genetic component. Moreover, the association between the presence of polymorphisms of the IL-10 promoter with CNO pathogenesis has been reported [10]. It has been also demonstrated that mutation of the *pstpip2* gene in mice results in an autoinflammatory disease very similar to human CNO [8]. However, candidate genes including *PSTPIP1*, *CARD15/NOD2*, and *IL1RN*, were not associated with CNO in humans when analyzed in small cohorts [11, 12].

Cox et al. recently showed that recessive mutations in *FBLIM1* contribute to the pathogenesis of CNO [9]. *FBLIM1* codes for Filamin-binding LIM protein 1 (FBLP1 or migfilin), a filamin-binding protein involved in the regulation of bone remodeling [13].

FBLP1 is a key regulator of the cytoskeleton, as it is recruited to cell-matrix contacts in response to adhesion and colocalizes with beta-catenin at cell-cell junctions in epithelial and endothelial cells. Through interactions with multiple binding partners, including filamin, FBLP1 links the cell adhesion structures to the actin cytoskeleton [14]. FBLP1 competes with integrin β for filamin binding to promote integrin activation in neutrophils as well as bone homeostasis [15]. Therefore, a mutation in the filamin-binding domain of FBLP1 may disrupt FBLP1-FLN binding, resulting in aberrant integrin activation in neutrophils and leading to sterile inflammation.

In our cohort of 80 CNO patients we found a rare coding variant of *FBLIM1* in 18 patients. Three of these variants (rs146575757, rs41310367 and rs114077715) were previously described by Cox et al. [6]. The missense variant Arg38Gln (rs146575757) is in the filamin-binding domain (exon 3), so it may be causative of neutrophil activation. The second variant (rs41310367) is centrally located in the middle of an enhancer, in a STAT3 binding region and in an NR4A2 recognition site, reported to be active in several cell lines, including osteoblasts, so it may disrupt balance between osteoclasts and osteoblasts activity leading to bone remodeling. The other missense variant Gly311Arg on the last exon is localized in the third LIM domain of the protein, a small protein -protein interaction domain, containing two zinc fingers.

For the further eight variants identified in our study no literature data are available about their association with the disease or their functional impact. These variants are all rare (MAF < 2%) in the general population, and extremely rare in the case of rs540511146 (MAF = 0.00011). The high prevalence of *FBLM1* rare variants in our CNO cohort may support a role in the pathogenesis of the disease.

Considering the intronic variants, they were predicted to possibly alter binding sites for splicing factors, in particular for the serine/arginine-rich protein (SR) specific binding sites. We observed by Human Splicing Finder a prediction in which the substitution of a single nucleotide in that specific genomic region leads to a loss and/or possible new sequence recognition by these factors and a possible creation a sequence for a new splice site. The in silico analysis and its predictions could address the research towards functional studies to verify the pathogenicity of the variants.

No statistical association was found between patients with and without *FBLIM1* gene variants, suggesting that *FBLIM1* might be considered a non-specific predisposing factor to CNO in a subgroup of patients.

## Conclusions

CNO remains a not completely understood disease but it probably belongs to the family of autoinflammatory diseases. A unique causing gene was not found yet. However, our data seem may support the fact that some *FBLIM1* variants might increase the susceptibility to the CNO pathogenesis.

## Supplementary information

**Additional file 1: Supplementary table**. Frequencies (Global, European, South Asian, East Asian and African) of *FBLIM1* gene variants in healthy control people (from GnomAD v2.1.1 controls dataset).

## Data Availability

The datasets used and/or analysed during the current study are available from the corresponding author on reasonable request.

## Abbreviation

CNO: Chronic Non-bacterial Osteomyelitis
