# Supplementary material for: High prevalence of rare FBLIM1 gene variants in an Italian cohort of patients with Chronic Non-bacterial Osteomyelitis (CNO)
Source: Pediatr Rheumatol Online J. 2020 Jul 10;18:55. doi: 10.1186/s12969-020-00447-4 (PMC7350626; doi:10.1186/s12969-020-00447-4)
Supplement: Supplementary file 1 — Additional file 1: Supplementary table. Frequencies (Global, European, South Asian, East Asian and African) of FBLIM1 gene variants in healthy control people (from GnomAD v2.1.1 controls dataset). [file 12969_2020_447_MOESM1_ESM.docx]

| **rs** | **Global** | **European** | **South Asian** | **East Asian** | **African** |
| --- | --- | --- | --- | --- | --- |
| rs114077715 | 0.02057 | 0.02829 | 0.01033 | 0.000 | 0.004420 |
| rs201006671 | 0.002399 | 0.001820 | 0.000 | 0.002188 | 0.002490 |
| rs76050903 | 0.002399 | 0.001820 | 0.000 | 0.002183 | 0.004297 |
| rs61733331 | 0.006850 | 0.001889 | 0.01355 | 0.006030 | 0.03931 |
| rs187479896 | 0.0007395 | 0.0009410 | 0.0008426 | 0.000 | 0.000 |
| rs140170023 | 0.01058 | 0.01615 | 0.006518 | 0.000 | 0.001989 |
| rs540511146 | 0.00009146 | 0.00002339 | 0.0005739 | 0.000 | 0.000 |
| rs766409425 | 0.00002920 | 0.00007467 | 0.000 | 0.000 | 0.000 |
| rs144567113 | 0.01327 | 0.01988 | 0.004424 | 0.000 | 0.001850 |
| rs146575757 | 0.003571 | 0.001896 | 0.01348 | 0.006183 | 0.001457 |
| rs41310367 | 0.02238 | 0.02813 | 0.03701 | 0.000 | 0.004635 |
| rs540511146 | 0.00009146 | 0.00002339 | 0.000 | 0.000 | 0.000 |

Supplementary table. Frequencies (Global, European, South Asian, East Asian and African) of *FBLIM1* gene variants in healthy control people (from GnomAD v2.1.1 controls dataset).
